# Supplementary material for: Impact of walking speed and motion adaptation on optokinetic nystagmus-like head movements in the blowfly Calliphora
Source: Sci Rep. 2022 Jul 7;12:11540. doi: 10.1038/s41598-022-15740-3 (PMC9262929; doi:10.1038/s41598-022-15740-3)
Supplement: Supplementary file 1 — Supplementary Information. [file 41598_2022_15740_MOESM1_ESM.pdf]

# Supplementary Material

## Impact of walking speed and motion adaptation on optokinetic nystagmus-like head movements in the blowfly *Calliphora*

Kit D. Longden, Anna Schützenberger, Ben J. Hardcastle, Holger G. Krapp

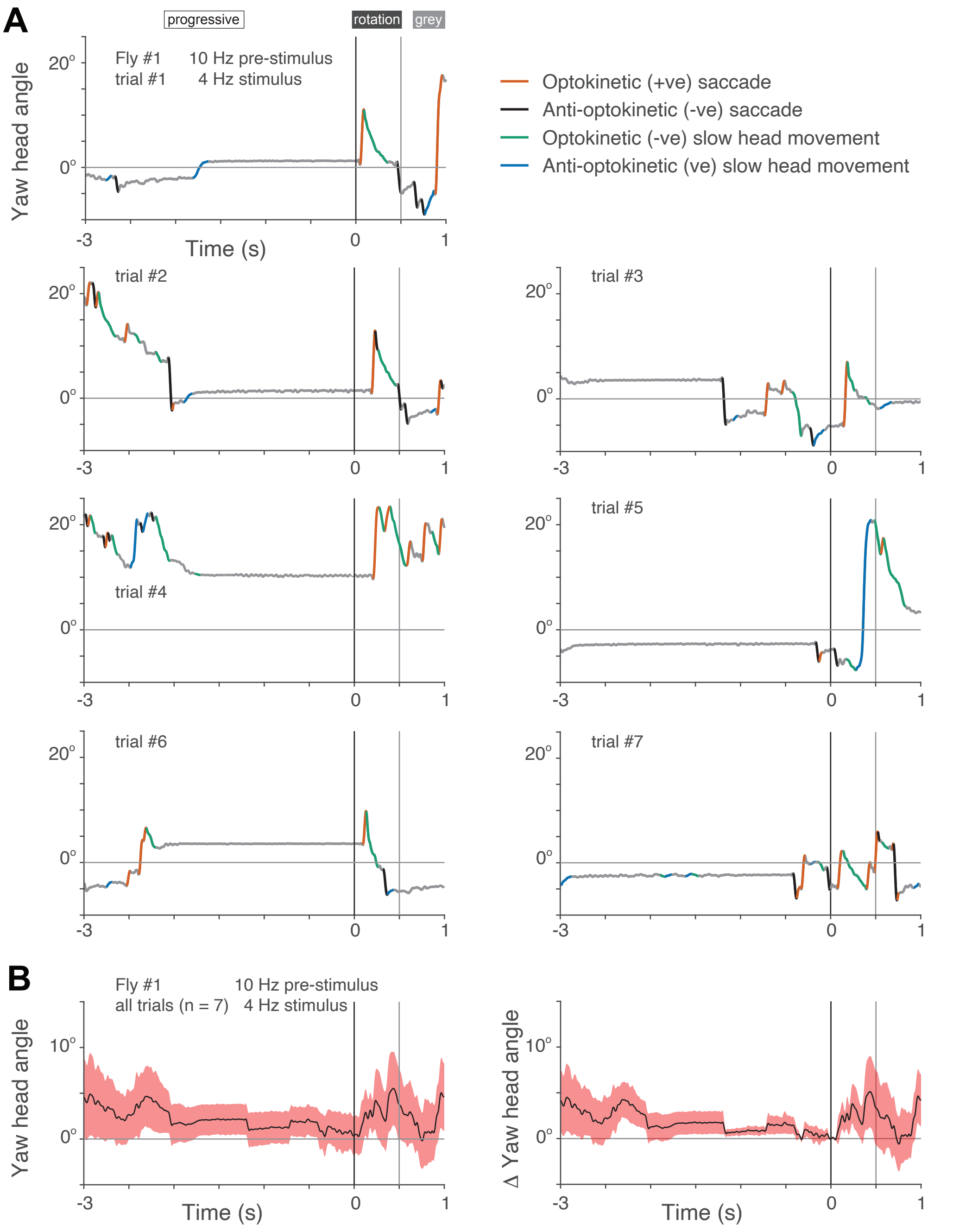


**Figure S1. Examples of head yaw movements and their classification. A.** All trials of fly #1 viewing 10 Hz progressive motion, followed by 4Hz rotational image motion in the stimulus period, for trials where the range of yaw head movement ≥ 2.5°. The turns are classified as saccades or slow turns using the duration and peak velocity of the turn (Fig. 2E: see Materials and Methods). The classification of individual turns are color coded, with unclassified turns in gray. Turns are unclassified when they are too slow to be classified as saccades, and too brief to be classified as slow head movements (Fig. 2E). This classification scheme emphasizes large and sustained head movements; inclusion of small, oscillatory head movements did not affect the conclusions of the analysis. Trial #2 is also displayed in Fig. 2A. Typically, the onset of rotational image motion is followed by a saccade against the direction of optic flow, then slow syndirectional head movements. However, other behaviors also occur, for example slow movements against the direction of motion (trial #5) and syndirectional saccades (trial #6). **B**. Mean ±S.E.M. head yaw angles of trials shown in A (left), and the change in the head yaw angle from the onset of the rotational image motion at t = 0 s (right), that offsets the effects of different initial head yaw positions on the mean. The sequences of a saccade against the direction of motion followed by a syndirectional slow head movement result in an average small positive head movement, that does not reveal the underlying optokinetic-like dynamics.

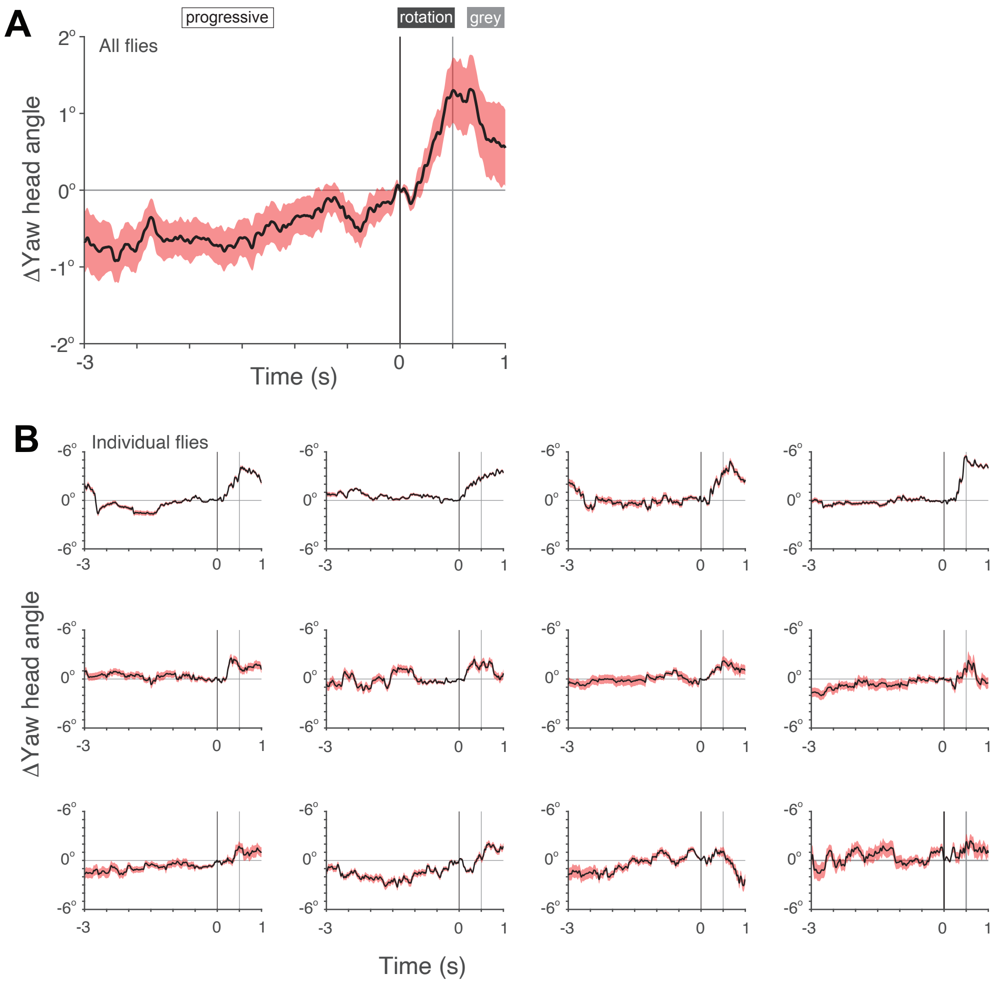


**Figure S2. Average head movement responses to rotational image motion.** A. Average changes in the yaw head angle from the onset of the rotational image motion of all flies for the pooled responses to all prestimulus conditions and 4, 7 and 10 Hz stimulus conditions, for trials with head movements (where the range of yaw head movements ≥ 2.5º); mean ±S.E.M. shown (N = 18). As for single prestimulus and stimulus conditions (Fig. S1B), the sequences of a saccade against the direction of motion followed by a syndirectional slow head movement result in an average small positive head movement that does not reveal the underlying optokinetic-like dynamics. **B**. Average changes in the yaw head angle from the onset of the rotational image motion of individual flies, for the pooled responses to all prestimulus conditions and 4, 7 and 10 Hz stimulus conditions, for trials with head movements (where the range of yaw head movements ≥ 2.5º), mean ±S.E.M. calculated over all trials.
